# CMV-associated encephalitis and antineuronal autoantibodies - a case report

**DOI:** 10.1186/1471-2377-12-87

**Published:** 2012-09-04

**Authors:** Xinling Xu, Peter Bergman, Thomas Willows, Charlotte Tammik, Marie Sund, Tomas Hökfelt, Cecilia Söderberg-Naucler, Stefania Varani

**Affiliations:** 1 Department of Medicine, Karolinska Institutet, Stockholm, Sweden; 2 Department of Laboratory Medicine F68, Karolinska University Hospital and Karolinska Institutet, Huddinge, Stockholm, Sweden; 3 Department of Neurology, Karolinska University Hospital Huddinge, Stockholm, Sweden; 4 Department of Radiology, Karolinska University Hospital Huddinge, Stockholm, Sweden; 5 Department of Neuroscience, Karolinska Institutet, Stockholm, Sweden; 6 Department of Hematology and Oncology, Section of Microbiology, University of Bologna, Bologna, Italy; 7 Department of Medicine, Center for Infectious Medicine (CIM), Karolinska University Hospital and Karolinska Institutet Huddinge, Stockholm, Sweden

**Keywords:** Human cytomegalovirus, Encephalitis, Antineuronal autoantibodies

## Abstract

**Background:**

Human cytomegalovirus (CMV) is an ubiquitous pathogen capable of modulating the host immune system. Immune dysfunction is common during CMV infection and includes autoimmune phenomena. Here we focus on a case of primary CMV infection associated with encephalopathy in a patient with a rudimentary spleen. We discuss diagnostic challenges and immunological aspects as well as the hypothesis that CMV may break tolerance and induce potentially encephalitogenic autoantibodies.

**Case presentation:**

A 33-year-old woman was admitted with features of encephalitis, rapidly progressing into a catatonic state. The patient tested negative for presence of herpes simplex virus DNA in cerebrospinal fluid (CSF), and had elevated liver enzymes and hepatomegaly at computed tomography scan (CT) examination. CT scan and magnetic resonance imaging (MRI) showed only a rudimentary spleen. Initially, serum was negative for anti-CMV IgM, but borderline for anti-CMV IgG by enzyme-linked immunosorbent assay. However, a more sensitive assay resulted in a positive specific IgM Western blot profile and low IgG avidity, suggesting primary CMV infection. Further, CMV DNA was retrospectively detected in a CSF sample collected at admission. We also detected antineuronal autoantibodies, which stained GAD-positive neurons in the hippocampus. The patient was treated by a combination of prednisone, intravenous immunoglobulins (IVIg) and antivirals, which resulted in a dramatic amelioration of the patient’s neurological status. One year after admission the patient exhibited a nearly complete recovery with mild deficits in attention and memory.

**Conclusions:**

A possible reason for the critical course of CMV infection could be the lack of a functional spleen in this patient, a condition previously associated with severe CMV infection. Prompt treatment with antiviral drugs, steroids and IVIg was most likely important for the positive outcome in this case and should be considered for similar cases of severe primary CMV infection associated with immunopathological phenomena.

## Background

Human cytomegalovirus (CMV) is an ubiquitous pathogen capable of modulating the host immune system. Immune dysfunction is common during CMV infection and include autoimmune phenomena [1,2] and Guillain-Barré syndrome [3], the latter suggesting that CMV may contribute to neuropathological processes. In patients with autoimmune diseases, the virus can replicate at sites of inflammation [4], highlighting CMV’s potential role in inducing or maintaining immunopathology. Here we focus on a case of primary CMV infection associated with encephalopathy in a patient with a rudimentary spleen. We discuss diagnostic challenges and immunological aspects as well as the hypothesis that CMV may break tolerance and induce potentially encephalitogenic autoantibodies. Finally, the clinical utility of antivirals combined with intravenous immunoglobulins (IVIg) and prednisone in the treatment of severe CMV infections is highlighted.

## Case presentation

**Figure 1:**
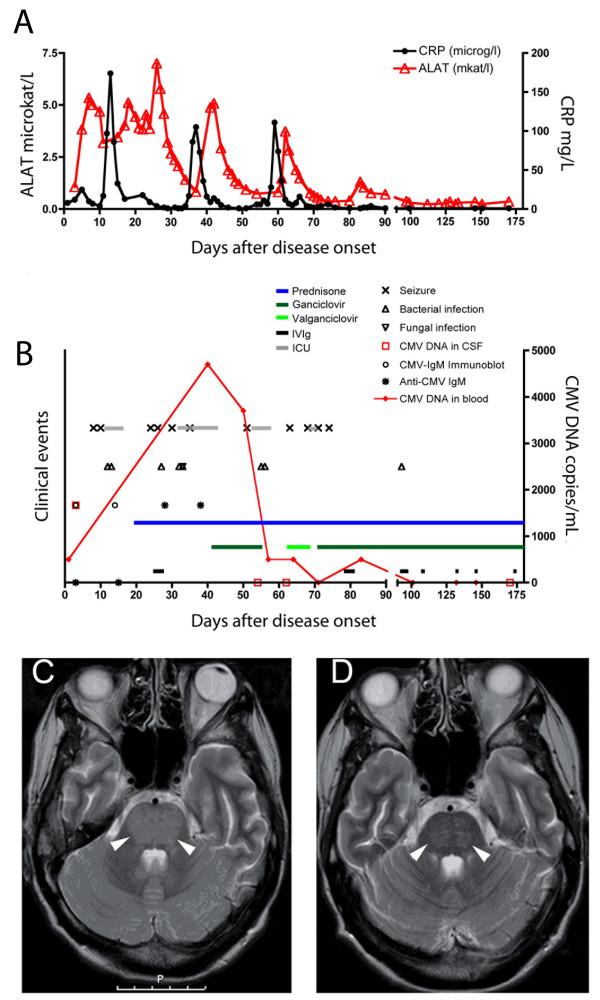
**(A) Laboratory values.** Normal ranges: C-reactive protein (CRP), <5 mg/L; alanine aminotransferase (ALAT), <0.75 microkat/L. (**B**) **Clinical course and treatment**. ICU; admission to intensive care unit. Prednisone was administered at 80 mg/day (starting at Week 3) and later adjusted to 60 mg/day in a lowering dose until complete drug withdrawal. Ganciclovir was started at Week 6 at 5 mg/kg per day. Antivirals were administered consecutively for 5 months, while IVIg (0.4 g/kg/day on 5 days) was administered on a three week-basis. (**C**) and (**D**) **Imaging findings**. Twenty days after disease onset, T2-weighted MRI scans showed increased signal in the pons (arrowheads), mesencephalon, globus pallidus, and external capsula while six months later (D) complete regression of the signal enhancement (arrowheads) and an intact blood–brain barrier were observed.

A 33-year-old woman presented to the emergency room at Karolinska University Hospital, Huddinge, Sweden, with a history of confusion, fatigue, impaired memory and headache. Symptom onset and duration was unclear; the patient had experienced an upper respiratory tract infection 10 days earlier. The medical history was unremarkable, except an episode of acute idiopathic thrombocytopenic purpura (ITP) 15 years earlier. After admission, the confusion progressed and the patient experienced mild speech difficulties in terms of response latency and difficulty in finding words. She also had memory impairment and motor agitation. The patient was swaying whilst performing the Romberg test and had tremors. Altered mental status with confusion and impaired memory aroused suspicion of limbic encephalitis. Initial blood tests including leukocyte count (9.5 x 10^9^/L) and C-reactive protein (Figure 1A) were normal. Cerebrospinal fluid (CSF) had a high concentration of albumin, as a sign of barrier injury, and normal cell count. The patient was admitted to the Neurology Department of the same hospital and empirically treated with acyclovir under suspicion of herpes simplex type I (HSV-I) encephalitis. As CSF tested repeatedly negative for HSV-1 DNA by a high sensitive real-time PCR [5], acyclovir was discontinued.

On Day 9 after admission, a short loss of consciousness was reported, and on Day 10 the patient was found unconscious, with tonic extensions/spasms of the extremities, involuntary loss of urine and tongue bites. Despite administration of Diazepam and Fosphenytoin, the patient remained unconscious and was transferred to the Intensive Care Unit (ICU) (Figure 1B). She thereafter entered a catatonic condition. Continuous electroencephalography (EEG) monitoring showed frontal bilateral sparse hints of sharp waves/epileptic activity but no certain seizure activity. After this episode, the patient had several seizures in form of tonic extensions/spasms of the extremities with autonomic influence and she was treated at the ICU at 4 more occasions. Repeated EEG examinations did not show any epileptic or seizure activity even when the patient experienced tonic extensions/spasms.

At Week 3, the patient presented myoclonic jerks in all four extremities with irregular pattern, which were triggered by auditory and sensory stimuli. Occasionally, the patient also exhibited ophistotonus. EEG examinations was performed while the patient exhibited these jerks, but no signs of epileptic changes or seizure activity were found, suggesting cortical myoclonus and brainstem evoked decerebration seizures.

Computed tomography (CT) and magnetic resonance imaging (MRI) head scans did not show any signs of cerebrovascular insult, or malignancy. Nevertheless, accumulation of contrast signal was detected by MRI in pons, brainstem, thalamus and in the cerebellar leptomeninges (Figure 1C), which was judged as a non-specific sign of inflammation.

The patient had elevated liver enzymes (Figure 1A and data not shown) and hepatomegaly at CT examination, which led to the suspicion of autoimmune liver disease. In support of this, the patient exhibited hypergammaglobulinemia and anti-nuclear, anti-smooth muscle cell and anti-SS-A52 autoantibodies. Several other autoantibodies were also detected, including anti-MitoM2, M2-3E, gp120, Sp100, PML and anti-mitochondrial autoantibodies indicating primary biliary cirrhosis as well as anti-Ro52, anti-Ro60, SS-B and anti-dsDNA antibodies as in Sjögren’s syndrome or systemic lupus erythematosus. However, a biopsy of the lip salivary gland was negative for histological hallmarks of Sjögren’s syndrome and no other systemic autoimmune disease could be confirmed.

CT scan and MRI showed only a rudimentary spleen. Notably, the patient’s former medical history of ITP did not involve splenectomy.

At Week 23 after disease onset the total T-cell count was normal (5x10^9^ cells/L), but low levels of CD4+ T cells and increased levels of CD8+ T cells were observed (38% and 87% of total T cells, respectively). The B-cell count was lower than normal and T- and B-cell proliferation upon mitogen stimulation was impaired (data not shown).

At admission, serum and CSF samples tested negative for varicella–zoster virus, borrelia, *Treponema pallidum,* and toxoplasma and revealed past contact for Epstein-Barr virus, adenovirus, and measles. Of note, a serum sample tested negative for anti-CMV IgM, but borderline for anti-CMV IgG by enzyme-linked immunosorbent assay. Because of the unclear result for specific IgG, a blood sample was analysed at Week 5 and tested positive for CMV DNA, indicating active CMV replication (Figure 1B). A CSF sample collected at admission was retrospectively analysed and CMV DNA was detected by PCR. Two serum samples, obtained from the patient at admission and ten days later, retrospectively showed a positive specific IgM profile by immunoblot analysis (method described in [6]) and low IgG avidity, suggestive of primary CMV infection.

Empirical treatment with intravenous high-dose cortisone was initiated during Week 3 (Figure 1B), followed by oral prednisone and intravenous immunoglobulins (IVIg) at Week 4. When the diagnosis of active CMV infection was confirmed, intravenous ganciclovir treatment was initiated in combination with prednisone; administration of antivirals and steroids was continued daily in a course of 5 months, while IVIg were administered in an intermittent way in nine doses (Figure 1B and data not shown). This therapy resulted in a dramatic amelioration of the patient’s neurological status. Radiological examinations of the brain showed regression of pathological signs (Figure 1). Liver enzymes normalized (Figure 1A). CMV DNA load decreased and then tested negative (Figure 1B). After four months, the patient was discharged to a rehabilitation ward. She remained on antivirals for a total of five months while prednisone was diminished on a scalar basis until complete discontinuation after 11 months. One year after admission the patient exhibited a nearly complete cognitive recovery with mild deficits in attention and memory. At neurological examination the only sequelae were oscillating eye movement at abduction, minor sporadic myoclonus and slightly impaired balance. The patient is now living by herself and attending college.

### Immunological findings

**Figure 2:**
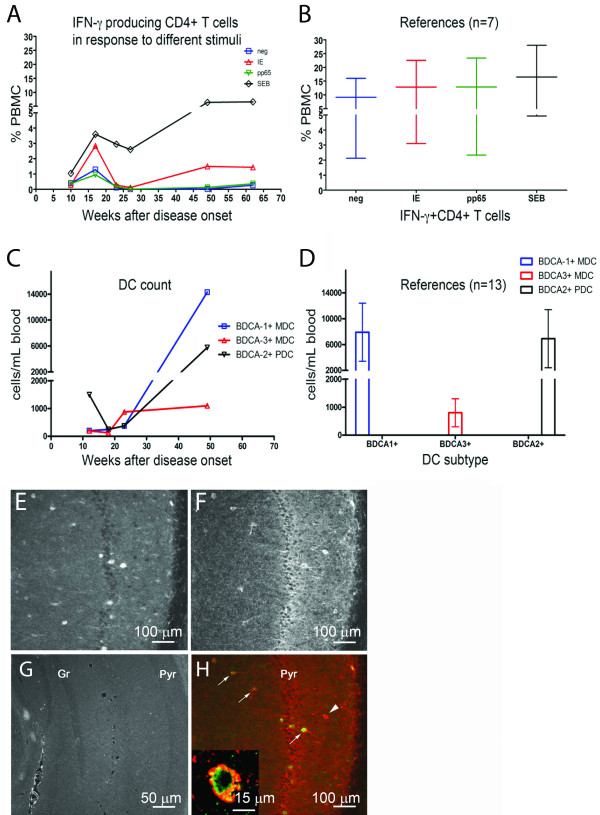
**(A,B) Percentages of CD4**^**+**^**T cells producing IFN-γ in response to CMV immediate early (IE) antigen-1 and pp65 in the patient (A) and 7 CMV-seropositive donors (B).***S. aureus* enterotoxin B (SEB) served as positive control. PBMC; peripheral blood mononuclear cells. (**C**,**D**) **Enumeration of DC subsets in the patient** (**C**) **and 13 donors** (**D**). BDCA; blood dendritic cell antigen. (**E**-**H**) **Neuroimmunological findings. **(**E**,**G**) Hippocampal neurons (granular [Gr] and pyramidal [Pyr] cell layers) after immunostaining with patient serum (green, 1:25,000) (E) or serum from control (1:5,000) (G). (F, H) Double-labeling with patient serum (green) and GAD (red). Arrows and inset indicate double-labelled cells. Arrowhead; GAD-positive serum-negative cell.

At Week 10, IFN-γ production by CD4+ and CD8+ T cells was low in response to CMV antigens. In addition, T-cell responses to *S. aureus* Enterotoxin B (SEB) were also low, indicating a general impairment of cellular immunity (Figure 2A and data not shown). CMV-specific CD4+ and CD8+ T-cell response, particularly to the immunodominant antigen pp65, was impaired up to Week 62 (Figure 2A and data not shown, method described in [7]). Conversely, high levels of IFN-γ were observed in plasma at Week 2 (499 pg/mL) and were maintained at Week 21 (154 pg/mL; reference values obtained from 13 healthy donors; 10 ± 28 pg/mL, mean ± standard deviation (data not shown).

At Week 12 low levels of BDCA-1+ (CD1c) and BDCA-3+ (CD141) myeloid dendritic cells (DCs) and BDCA-2+ (CD303) plasmacytoid DC were detected and remained impaired up to Week 24 (Figure 2C and D).

### Neuroimmunological findings

Since the patient presented with clinical signs of limbic encephalitis (cognitive impairment, seizures and pathologic MRI-scan), the presence of autoantibodies against antigens known to be involved in triggering this pathological condition was investigated [8]. Serum and CSF samples were negative for anti-Hu, anti-Ma, anti-voltage gated potassium channel, anti-collapsin response mediator protein-5 as well as for autoantibodies against the N-methyl-D-aspartate (NMDA) receptor. Further, we screened for the presence of neuronal autoantibodies, by applying patient serum to sections of murine brains as described [9]. A strong staining in neurons of cortex and hippocampus but not in glial cells was observed (Figure 2E and F). To assess the specificity of this finding, sera from 3 healthy CMV IgG-negative and 6 CMV IgG-positive controls were investigated; very weak or absent staining was observed (Figure 2G and data not shown). Interestingly, many – but not all – neurons in hippocampus that were stained by the patient serum were also immunoreactive for glutamic acid decarboxylase (GAD) (Figure 2H, inset).

## Discussion

Here, we describe a case of primary CMV infection associated with encephalitis and autoimmune phenomena in a young woman. This case was unusual for several reasons: i) CMV infections involving the central nervous system are uncommon in previously healthy individuals [10]; ii) an impaired CMV-specific T-cell response was observed for several months after infection; iii) the patient developed antineuronal autoantibodies directed against neurons in the hippocampus.

First, the patient exhibited a rudimentary spleen, which could have contributed to the lack of development of a proper anti-CMV immune response leading to severe infection. In fact, the spleen is crucial for development of IgM memory B-cells, which are important for immunity against viruses, including CMV [11] and splenectomised patients have been described to suffer from more severe CMV infections than eusplenic patients [12].

The poor IgM development against CMV in patients with a dysfunctional or absent spleen may complicate diagnostic procedures [11]. In our case, IgM against CMV tested negative during the active phase of infection by employing a commercial immunoenzymatic assay, which delayed the time to diagnosis. The diagnosis was eventually established through detection of circulating CMV DNA. Further, by the use of a more sensitive IgM Western blot method, IgM antibodies could be detected, which confirmed the diagnosis of active CMV infection.

The immunological status of the patient at disease onset showed low B-cell count as well as deficient lymphocyte proliferative response to mitogens. As a further sign of impaired immunity, circulating DC count was low; this is a common finding during primary symptomatic CMV infection in adults, as we recently reported [13]. Cytotoxic T lymphocytes (CTL) appear to play an important role in the control of CMV infection, 70-90% of all CTL being specific for the viral antigen pp65 [14]. Importantly, CD4+ and CD8+ T-cell responses to pp65 remained impaired up to Week 62 in our patient, while high plasma levels of IFN-γ suggest activation of innate mechanisms to compensate for the lack of CMV-specific responses. We hypothesize that uncontrolled viral replication in a subject with previously hidden immune-defects may have caused the virus to reach the central nervous system, thus triggering viral encephalitis. However, we cannot exclude that the virus *per se* may have contributed to impeding the host response. In fact, it is known that CMV inhibits antigen presenting cell function leading to impaired T cell responses (as reviewed in [15]) and alters mitogen-induced lymphocyte proliferation [16,17].

A third unusual feature of this case is that we detected autoantibodies with strong specificity to neurons; primary CMV infection may have contributed to the break of self-tolerance and to a broad polyclonal activation of B-cell clones with the final result of several autoantibodies, including those binding to neurons. Many neuronal antibodies stained cells that were GAD-positive, suggesting that the GABAergic system might be involved in the development of the observed immunopathology as anti-GAD autoantibodies have been linked to cerebellar ataxia and seizures [18]. However, since not all serum IgG-positive cells expressed GAD, other autoantigen(s) may be involved in the actual case.

A key observation that links active CMV infection and viral-induced immunopathology to the overall clinical presentation is the fact that the onset of a combined treatment including antivirals, immunomodulatory IVIg and prednisone coincided with a rapid improvement of the patient’s clinical status (Figure 1B). Therapeutical effect of single drugs could not be discerned, as ganciclovir and immunomodulatory drugs were administered simultaneously. It is known that antivirals are effective in the management of direct effects caused by CMV replication in immunocompromised patients [4], while optimal therapy for treating virus-induced immunopathology remains obscure; it is possible that IVIg and prednisone contributed to the positive outcome in this case.

## Conclusion

The patient described here had a primary CMV infection, which was associated with clinical and radiological signs of encephalitis. A possible reason for the critical course of CMV infection could be the lack of a functional spleen in this patient, a condition previously associated with severe CMV infection. Supporting this possibility, we observed poorly responding T cells to CMV antigens which lasted several months after the infection’s onset. CMV encephalitis in immunocompetent individuals has been associated with mild signs of inflammation of the CNS, including absent to moderate pleocytosis [19-21]. Our patient exhibited normal cell count and the presence of CMV DNA in the CSF at disease’s onset. Our main hypothesis is that primary CMV infection was the first insult and had a fulminant course because of the patient’s immune status (asplenic). Tolerance was broken and autoimmunity ensued. Infection of the CNS and general inflammation may have injured the blood brain barrier, thus allowing pathogenetic serum antibodies to enter the brain and cause neuronal damage. In support to our hypothesis, the need of an inflammatory trigger to allow serum antibodies to bind neurons in the brain has been demonstrated in a murine model of lupus [22]. Because autoantibodies binding to GAD-positive neurons were detected in this patient, we propose that the emerging concept of autoantibody-mediated encephalitis should be included in the differential diagnostic algorithm for patients presenting with signs of viral encephalitis as the two conditions may overlap. Prompt treatment with antiviral drugs, steroids and IVIg was most likely important for the positive outcome in this case and should be considered for similar cases of severe primary CMV infection associated with immunopathological phenomena.

## Consent

Written informed consent was obtained from the patient for publication of this Case report and any accompanying images. A copy of the written consent is available for review by the Series Editor of this journal.

## Abbreviations

BDCA, Blood Dendritic Cell Antigen; CMV, Cytomegalovirus; CSF, Cerebrospinal Fluid; CT, Computed Tomography; DC, Dendritic Cell; GAD, Glutamic Acid Decarboxylase; HSV, Herpes Simplex Virus; ITP, Idiopathic Thrombocytopenic purpura; IVIg, Intravenous Immunoglobulin; MRI, Magnetic Resonance Imaging.

## Competing interests

The authors declare no conflict of interest.

## Authors’ contributions

Collecting clinical data: XX, TW, MS, SV. Patient care: MS, TW. Immunological analyses: CT, CSN, SV. Neuroimmunological analyses: PB, TH. Writing manuscript: XX, PB, TH, CSN, SV. All authors read and approved the final manuscript.

## Authors' information

XX: MD, PhD-student, Currently doing her internship; PB: MD, PhD, PostDoc, Residency in Clinical Microbiology; TW: MD, Specialist in Neurology; CT: MSc, LabManager, Immunology; MS: MD, Residency in Radiology; TH: MD, PhD, Professor emeritus in Neuroanatomy; CS-N: MD, PhD, Professor in Microbial Pathogenesis; SV: MD, PhD, Assistant Professor, Specialist in Microbiology and Virology.

## Pre-publication history

The pre-publication history for this paper can be accessed here:

http://www.biomedcentral.com/1471-2377/12/87/prepub
